# Assessment of Kidney Function Discrepancies in Pediatric CAKUT Patients Using Bedside Schwartz Equation and Renal Scintigraphy

**DOI:** 10.3390/diseases12110265

**Published:** 2024-10-23

**Authors:** Ruxandra Maria Steflea, Geethiikha Jammula, Akhila Kanka, Caius Glad Streian, Felix Bratosin, Avram Cecilia Roberta, Monica Susan, Octavia Oana Harich, Casiana Boru, Sonia Tanasescu, Dan-Mihai Cristescu, Andreea-Mihaela Banta, Gabriela Doros, Bogdan Feciche

**Affiliations:** 1 Department of Pediatrics, “Victor Babes” University of Medicine and Pharmacy, 300041 Timisoara, Romania; steflea.ruxandra@umft.ro (R.M.S.); tanasescu.sonia@umft.ro (S.T.); doros.gabriela@umft.ro (G.D.); 2 “Louis Turcanu” Emergency Hospital for Children, 300011 Timisoara, Romania; 3 Doctoral School, “Victor Babes” University of Medicine and Pharmacy Timisoara, 300041 Timisoara, Romania; andreea.banta@umft.ro; 4 Faculty of Medicine, Dr. NTR University of Health Sciences, Ramavarappadu, Vijayawada 520008, Andhra Pradesh, India; geethiikhaj@gmail.com (G.J.); akhi47712@gmail.com (A.K.); 5 Department of Cardiac Surgery, “Victor Babes” University of Medicine and Pharmacy Timisoara, 300041 Timisoara, Romania; 6 Department of Infectious Disease, “Victor Babes” University of Medicine and Pharmacy, 300041 Timisoara, Romania; felix.bratosin@umft.ro; 7 Department of Residential Training and Post-University Courses, “Vasile Goldis” Western University, 310414 Arad, Romania; avram.cecilia@uvvg.ro; 8 Clinical Analysis Laboratory, Clinical Emergency Hospital of Arad County, 310037 Arad, Romania; 9 Department of Internal Medicine I, Centre for Preventive Medicine, “Victor Babes” University of Medicine and Pharmacy, 300041 Timisoara, Romania; susan.monica@umft.ro; 10 Department III of Functional Sciences, Discipline of Physiology, “Victor Babes” University of Medicine and Pharmacy, 300041 Timisoara, Romania; harich.octavia@umft.ro; 11 Department of Medicine, “Vasile Goldis” University of Medicine and Pharmacy, 310414 Timisoara, Romania; boru.casiana@uvvg.ro; 12 Research Centre of Timisoara Institute of Cardiovascular Diseases, “Victor Babes” University of Medicine and Pharmacy, 300041 Timisoara, Romania; dan.cristescu@umft.ro; 13 Department of Urology, Emergency County Hospital Oradea, Strada Gheorghe Doja 65, 410169 Oradea, Romania; feciche.bogdanovidiu@didactic.uoradea.ro

**Keywords:** pediatric nephrology, chronic kidney disease, kidney imaging, kidney function assessment, renal scintigraphy

## Abstract

Background and Objectives: This research explores the correlation between estimated glomerular filtration rates (eGFR) obtained using the bedside Schwartz equation, and renal scintigraphy in children with congenital kidney and urinary tract abnormalities (CAKUT). The objective is to enhance understanding and management of renal health in this demographic by analyzing kidney size-function relationships. Methods: A retrospective observational analysis was performed on 94 pediatric CAKUT patients at the “Louis Turcanu” Emergency Hospital for Children, Timisoara. Kidney function data, extracted from medical records, were evaluated using the Schwartz equation, renal scintigraphy, and the gold standard iohexol clearance. Ethical approval was secured for the study, which employed descriptive and inferential statistical methods, including *t*-tests and correlation coefficients, to compare eGFR values. Results: Significant variances were found in eGFRs across different body surface area (BSA) percentiles. For instance, the eGFR for the right kidney in the 25th–50th BSA percentile (102.02 ± 41.52 mL/min/BSA) was notably higher than that of the left (35.60 ± 26.05 mL/min/BSA; *p* = 0.01). The overall sample reflected a higher eGFR in the right kidney (76.03 ± 40.91 mL/min/BSA) compared to the left (57.46 ± 35.91 mL/min/BSA; *p* = 0.02). Additionally, a strong positive Pearson correlation (r = 0.80, *p* = 0.02) was found between scintigraphy and ultrasound measures in the 50th–75th percentiles for left renal percentiles, demonstrating consistent patterns across different evaluations of kidney function. Conclusions: This comparison indicates a complex relationship between eGFR values and kidney size, suggesting potential inaccuracies in standard bedside eGFR measurements for pediatric CAKUT patients. The findings underscore the necessity for accurate diagnostic tools specifically designed for pediatric applications and advocate for the integration of multiple diagnostic techniques to improve clinical management.

## 1. Introduction

Ensuring optimal kidney function in children is paramount for their overall health and well-being [1]. Early detection can significantly impact a child’s life trajectory [2,3]. Among the myriad methods available for evaluating renal function, two widely utilized techniques stand out: the estimation of glomerular filtration rate (eGFR) through the bedside Schwartz equation, and renal scintigraphy [4,5]. Each method offers unique insights into renal health and function, yet understanding their comparative efficacy is crucial for clinicians striving to make informed decisions regarding patient care [1,6].

The comparison between the bedside Schwartz equation and renal scintigraphy, two well-established techniques for evaluating kidney function, forms the basis of our investigation. The bedside Schwartz equation, a widely used formula, offers a non-invasive means for eGFR, a key indicator of kidney health, by utilizing a child’s serum creatinine level and height [7,8,9]. However, factors like muscle mass and dietary habits can influence the equation’s accuracy [10].

On the other hand, renal scintigraphy provides a more definitive measurement of eGFR. This imaging technique employs a radioactive tracer to directly visualize and quantify the rate of blood flow through the kidneys [11]. While offering a more precise assessment, it involves radiation exposure and necessitates specialized equipment, making it less readily available compared to the bedside equation [11,12,13].

The size discrepancy between the two kidneys in children with vesicoureteral reflux and recurrent urinary tract infections is associated with the likelihood of an abnormal DMSA scan. This information could be valuable in determining the cost-effectiveness of subjecting a patient to an irradiating therapy [14]. Efficient surveillance of development and prompt interventions are vital to avert and manage growth deficiency in children with chronic kidney disease (CKD). Children may see a significant decrease in height throughout percentiles, particularly during periods of rapid growth [15,16].

This study takes the evaluation a step further by incorporating kidney size measurements obtained from the ultrasound. We will compare these measurements with established reference ranges for healthy kidney size in children, considering both height and body surface area in comparison with data provided by scintigraphy. This analysis aims to explore potential correlations between the physical size of the kidneys and their functional capacity as reflected by eGFR.

This study hypothesizes that the bedside Schwartz equation may have limitations in accurately estimating glomerular filtration rate (eGFR) in pediatric patients with congenital abnormalities of the kidney and urinary tract (CAKUT) due to their unique structural anomalies. The aim is to evaluate the accuracy of the Schwartz equation by comparing its eGFR estimations, not only against the renal scintigraphy using Tc-99m-DTPA but also against iohexol clearance, which is considered the gold standard. Additionally, the study seeks to investigate the relationship between kidney size (measured via ultrasound) and function (assessed through both scintigraphy and iohexol clearance) to determine if size–function correlations can enhance the understanding and management of kidney health in children with CAKUT.

## 2. Methodology

### 2.1. Study Design

A retrospective observational design was utilized in this investigation, wherein data were collected from the medical records of patients diagnosed with congenital abnormalities of the kidney and urinary tract. The research gained ethical approval from the Ethics Committee of “Louis Turcanu” Emergency Hospital for Children Timisoara (approval number 118/25.11.2023) and from “Victor Babes” University of Medicine and Pharmacy Timisoara (approval number 68/03.10.2022).

### 2.2. Patient Population

To determine the appropriate sample size for this study, several factors were considered: effect size, significance level (α), power, and variability. Using α = 0.05 and a power of 0.80, and considering the standard deviation of the scintigraphy measurements (56.37 mL/min/BSA), a simplified calculation suggested that a minimum of approximately 20 participants would be needed to detect a significant correlation.

The research includes a cohort of pediatric patients who had been previously diagnosed with CAKUT through abdominal imaging.

The term “CAKUT” refers to a collection of developmental abnormalities that impact the kidneys, encompassing changes in renal agenesis, size, location, and morphology, as well as the outflow tracts, including the ureters, bladder, and urethra. The outflow anomalies included ureteropelvic junction blockage, vesicoureteral reflux, duplex collecting system, megaureter, and posterior urethral valves [17].

Chronic kidney disease is characterized by the persistent presence of irregularities in the structure or function of the kidneys for a minimum duration of three months, which can have significant consequences for one’s general well-being. Our patients were classified as having CKD based on the structural abnormalities seen by imaging, which were found to be associated with their eGFR [2].

The staging of CKD was determined according to the recommendations provided by Kidney Disease: Improving Global Outcomes (KDIGO) [18]. The following categories were considered:

o GFR Category:
  ▪ G1 is defined as GFR ≥ 90 mL/min/1.73 m^2^;
  ▪ G2 is defined as GFR 60–89 mL/min/1.73 m^2^;
  ▪ G3a is defined as GFR 45–59 mL/min/1.73 m^2^;
  ▪ G3b is defined as GFR 30–44 mL/min/1.73 m^2^;
  ▪ G4 is defined as GFR 15–29 mL/min/1.73 m^2^;
  ▪ G5 is defined as GFR < 15 mL/min/1.73 m^2^;
o Albuminuria using albumin to creatinine ratio (ACR- approximate equivalent) category:
  ▪ A1 is defined as < 3 mg/mmol or < 30 mg/g;
  ▪ A2 is defined as 3–30 mg/mmol or 30–300 mg/g;
  ▪ A3 is defined as 30 mg/mmol or >300 mg/g.

The individuals hospitalized at “Louis Turcanu” Emergency Hospital for Children Timisoara were identified using electronic medical records from the period spanning June 2018 to May 2023.

The study ensured the preservation of patient confidentiality and privacy by de-identifying all data before analysis. In every instance, informed consent was acquired throughout the process of obtaining admission chart consent.

The eligibility criteria encompassed pediatric patients aged 0 to 18 who were hospitalized with CAKUT and underwent scintigraphy.

The exclusion criteria were the following:

- Children presenting with urinary tract infections during the assessment;
- Children who did not undergo scintigraphy concomitant with the measurement of creatinine levels;
- Children without informed consent from the caregivers for performing scintigraphy.

### 2.3. Data Collection

Patient records were analyzed to obtain demographic data, such as age, sex, environment of origin, weight, and height. Using CDC growth charts, we established height and weight percentiles and calculated the BSA using the formula: BSA [m^2^] = 0.024265 × H^0.03964^ [cm] × W^0.5378^ [kg] [19,20]. A comprehensive collection of clinical data, including diagnosis, comorbidities, and medication history, was obtained from all the included children.

The study includes laboratory tests of serum creatinine. Serum samples were analyzed using Cobas Integra^®^ 400 Plus machine by Roche Diagnostics. All values were provided by the internal laboratory. Additionally, urine samples were obtained from each patient and examined for the presence of proteinuria.

Renal scintigraphy using Tc-99m Diethylenetriamine pentaacetate (Tc-99m-DTPA) was performed within the nuclear medicine compartment of the “Pius Brinzeu” Timisoara County Clinical Emergency Hospital in the same admission when the serum creatinine value was collected.

The choice of DTPA (diethylenetriaminepentaacetic acid) over Cr-EDTA (chromium-ethylenediaminetetraacetic acid) for renal scintigraphy in our study is based on several factors including patient safety and the specific diagnostic needs of the pediatric population. DTPA is labeled with Technetium-99m, which has a shorter half-life, resulting in lower radiation exposure—a critical consideration in children. Moreover, DTPA is exclusively excreted by glomerular filtration, offering a clear and direct assessment of renal function. While both agents are effective at measuring glomerular filtration rate, the characteristics of DTPA align better with our study’s requirements for accuracy and safety in pediatric patients.

The procedure was performed after adequate hydration before or during the examination, which is essential for achieving optimal post-furosemide diuresis and preventing dehydration. The standard dose of furosemide is 1 mg/kg, with a suggested maximum dose of 40 mg [21]. The images were acquired with the patient supine on the imaging table.

An Esaote MyLab™Seven ultrasound machine was used to conduct abdominal ultrasonography. The MyLab™Seven, manufactured by Esaote, complies with the Medical Device Directive (MDD) 93\42\EEC. The investigation was carried out by trained pediatric radiologists or a pediatric nephrologist with ultrasonography competence and more than five years’ experience. The patients were examined in a supine position. Following the identification of the kidney, it was observed along its longitudinal axis, which intersected the kidney hilum. The measured values of kidney length in millimeters were documented for each individual. Following the acquisition of kidney length measurements, the percentiles of kidney length were determined in proportion to the body surface area and height of the patients at the time of the assessment [22].

### 2.4. Estimation of Glomerular Filtration Rate (eGFR)

Iohexol clearance was introduced as the gold standard for assessing glomerular filtration rate. Iohexol, a non-ionic, water-soluble radiographic contrast agent was administered intravenously, and its plasma clearance was measured to accurately determine GFR. This method served as a benchmark to evaluate the performance of the bedside Schwartz equation and Tc-99m-DTPA scintigraphy, both commonly used yet potentially less accurate in pediatric patients with congenital abnormalities of the kidney and urinary tract

The estimated glomerular filtration rate was determined using the bedside Schwartz formula, which takes into account serum creatinine levels and individual characteristics such as age, height, and weight. The formula is 0.413 multiplied by the ratio of height to serum creatinine (S_Cr_), where height is measured in centimeters and S_Cr_ is measured in mg/dL [23].

The eGFR was calculated using scintigraphy with the Gates method. This method estimates the total and individual kidney GFR by measuring the integral activity accumulated by each kidney during 120–180 s after injection. It assumes that all the glomerular filtrate produced after injection is still in the nephrons and has not yet left the kidney. In order to determine quantitative GFR values, the total counts were adjusted to account for the administered activity dose and time interval. The resultant values are characterized as clearance equivalent and were translated to individual and total quantitative renal clearance values given in milliliters per minute. The quantitative GFR is calculated by multiplying the regression coefficient (9.75) by the difference between the total renal uptake percentage and the intercept value (6.19) used in the Gates approach [24,25].

A comparative analysis was performed to evaluate the agreement and disagreement between the eGFR obtained from the two approaches mentioned above.

### 2.5. Statistical Analysis

The data pertaining to identification, as well as clinical and paraclinical factors, were meticulously recorded in a secure computerized database utilizing Microsoft Excel^®^ version 2312 (Build 17126.20132), a version which was released on 9 January 2024. The statistical analysis was conducted using MedCalc^®^ Statistical Software version 22.017, developed by MedCalc^®^ Software Ltd. in Ostend, Belgium. The software is accessible at https://www.medcalc.org and was accessed on 15 January 2024.

Patient demographics, clinical characteristics of each type of CAKUT, laboratory data, scintigraphy results, and ultrasound features were summarized using descriptive statistics. The distribution of the displayed data was evaluated using the Shapiro–Wilk test, and non-parametric statistical techniques were adopted due to an important deviation from a normal distribution. Correlation coefficients and Bland–Altman analysis were employed to evaluate the concordance between glomerular filtration rate estimations derived from the improved bedside Schwartz formula and renal scintigraphy. For variables that did not conform to a parametric distribution, measures of central tendency were calculated using medians and the inter-quartile range (IQR). In the case of parametric variables, the mean ± standard deviation was utilized. In this study, the correlation coefficient, Pearson correlation coefficient (r), was employed to evaluate the magnitude and direction of the relationships between ultrasound observations and the glomerular filtration rate provided by renal scintigraphy. The study also compared the estimated GFR from the Schwartz equation and Tc-99m-DTPA scintigraphy to the GFR derived from iohexol clearance. The Pearson correlation coefficient was calculated to quantify the degree of linear correlation between the measurements obtained from the different methods.

## 3. Results

### 3.1. Baseline Characteristics

This study included 94 children diagnosed with CAKUT. More than half of the children in the study were male (57.44%). More than half (55.31%) of patients (52) originated from rural areas. The median age at diagnosis was 24 months with a range of 2 to 93.50 months. The median age at the time of the scintigraphy examination, a diagnostic test often used in CAKUT, was 112 months, with a range from 61 to 147 months. The diagnosis was made either during the antenatal period or at birth, with the highest age of diagnosis being 204 months.

The median weight was 28 kg, ranging from 19 to 49.25 kg, and most children were within the normal weight range for their age, as indicated by the median weight percentile of 58. The median height was 136 cm, with a range from 110.87 to 150 cm, and most children were within the normal height range for their age, as indicated by the median height percentile of 65. The median body surface area was 1.02 m^2^, with a range from 0.77 to 1.43 m^2^. The baseline features of the sample undergoing study are presented in Table 1.

### 3.2. Types of CAKUT

Table 2 displays the types of CAKUT that have been found within our lot. In our cohort, compensatory hypertrophy was found in 19.15% of the patients, mainly those with renal hypoplasia or solitary kidney. Vesicoureteral reflux was observed in 27.65% of the patients. Out of these patients, 38.46% had right vesicoureteral reflux and 46.15% had bilateral vesicoureteral reflux.

### 3.3. Chronic Kidney Disease Staging

The incidence of chronic kidney stage among the children under analysis is presented in Table 3. Based on the albuminuria staging, all patients were in A1 category.

### 3.4. GFR Estimation Using Serum Creatinine and Scintigraphy

The analysis of Table 4 indicates that the estimated GFR (eGFR) from the Schwartz equation significantly underestimates the true GFR measured by iohexol plasma clearance, with a mean difference of −15.5 mL/min/1.73 m^2^ (*p* = 0.023). Despite this underestimation, there is a strong positive correlation between the Schwartz eGFR and iohexol GFR (r = 0.86, *p* < 0.001), suggesting consistent proportionality in the measurements. In contrast, the GFR obtained from renal scintigraphy closely aligns with the iohexol clearance, showing a minimal and statistically non-significant mean difference of +3.4 mL/min/1.73 m^2^ (*p* = 0.57). Furthermore, renal scintigraphy exhibits a very strong positive correlation with the gold standard (r = 0.91, *p* < 0.001), reinforcing its reliability. These findings suggest that, while the Schwartz equation tends to underestimate GFR, potentially limiting its accuracy in pediatric CAKUT patients, renal scintigraphy provides GFR measurements that closely reflect the true renal function as determined by the gold standard iohexol plasma clearance.

The bedside Schwartz technique yielded a median eGFR value of 115 mL/min, with an IQR ranging from 90.50 mL/min to 135 mL/min. The scintigraphy had an average value of 133.50 ± 56.37 mL/min/BSA. The scintigraphy of the right kidney had an average value of 76.03 ± 40.91 mL/min/BSA, while the left kidney had an average value of 57.46 ± 35.91 mL/min/BSA.

The differences between the two methods were plotted, and the arithmetic mean of these differences was found to be −24.33, with a 95% confidence interval ranging from −36.84 to −11.83. The *p*-value for the null hypothesis that the mean difference is zero was found to be 0.0003, which is statistically significant. The lower limit of the differences was −107.80, with a 95% confidence interval from −129.32 to −86.28. The upper limit was 59.13, with a 95% confidence interval from 37.61 to 80.65.

A regression analysis was performed to model the relationship between the two methods. The resulting regression equation was y = 35.49 − 0.49x, where y represents the eGFR from Method B (Scintigraphy), and x represents the eGFR from Method A (Creatinine).

**Figure 1:**
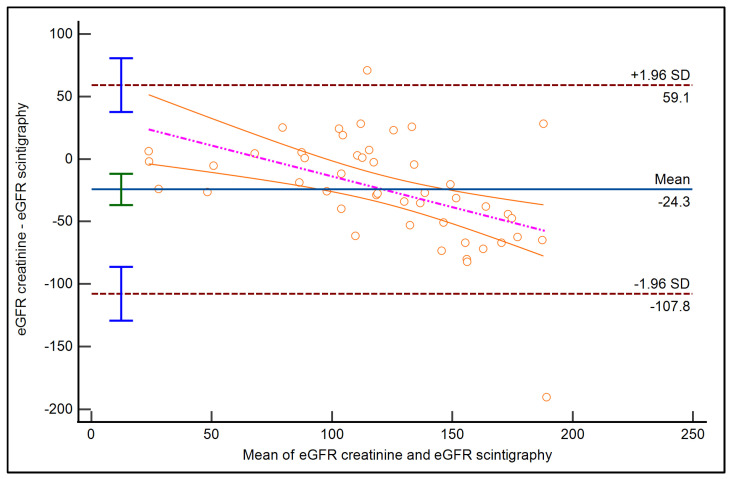
The graphical representation of the Bland–Altman plot is represented using the following parameters: a red dotted line representing the line of equality, a green interval representing the 95% confidence interval for the mean difference, blue intervals representing the 95% confidence interval for the upper and lower limits of agreement, and a purple dotted line representing the regression line of differences, with 95% confidence intervals represented by orange lines.

The intercept of the regression equation was 35.49 (*p* = 0.03, 95% CI: 2.07 to 68.92), and the slope was −0.49 (*p* = 0.0004, 95% CI: −0.75 to −0.23). The negative slope indicates that, as the eGFR from Method A increases, the eGFR from Method B decreases, suggesting a potential inverse relationship between the two methods. The *p*-values for both the intercept and slope are less than 0.05, indicating that they are statistically significant (Figure 1).

Table 5 displays the results of independent samples t-tests comparing estimated glomerular filtration rates from scintigraphy assessments of both kidneys across different body surface area percentiles. The data include mean values with standard deviations. A notable finding is in the 25th–50th BSA percentile range, where the right kidney shows a significantly higher eGFR (102.02 ± 41.52) compared to the left kidney (35.60 ± 26.05), with a statistically significant *p*-value of 0.01. Similarly, for the entire lot regardless of BSA percentile, the right kidney’s eGFR (76.03 ± 40.91) is significantly higher than that of the left (57.46 ± 35.91), with a *p*-value of 0.02. However, no significant differences were found in the other percentile ranges, indicating that substantial variations in kidney function as measured by eGFR may be associated with different BSA percentiles, particularly in the mid-range of BSA.

### 3.5. Correlation Between Renal Scintigraphy and eGFR

**Figure 2:**
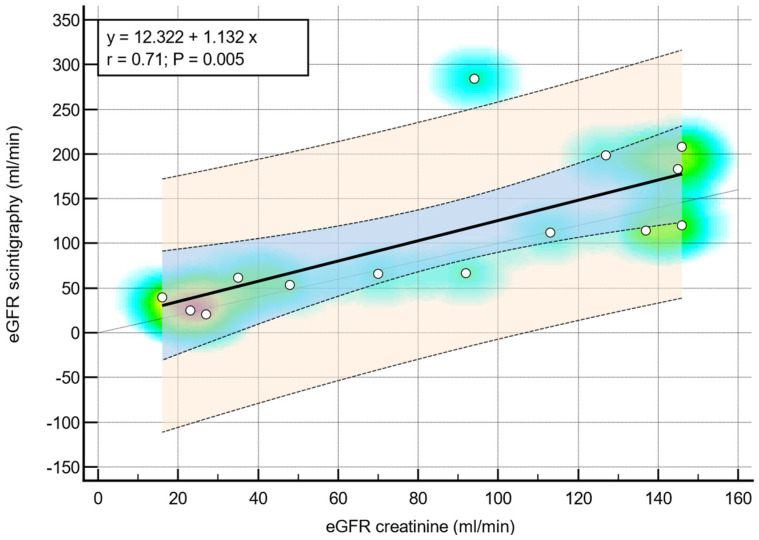
Strong positive linear correlation between eGFR scintigraphy and eGFR creatinine in children with CAKUT on both sides—scatter diagram with heat map. The background color coding indicates density of points, suggesting clusters of observations. The red color indicates a high concentration of points, yellow indicates a moderate concentration of points, and blue indicates a low concentration of points.

**Figure 3:**
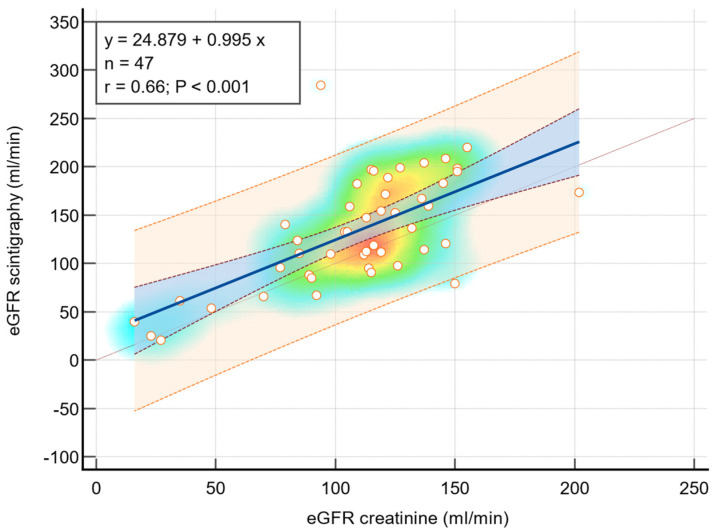
Strong positive linear correlation between eGFR scintigraphy and eGFR creatinine for all data analyzed—scatter diagram with heat map. The background color coding indicates density of points, suggesting clusters of observations. The red color indicates a high concentration of points, yellow indicates a moderate concentration of points, and blue indicates a low concentration of points.

The regression analysis conducted to assess the relationship between renal scintigraphy and estimated Glomerular Filtration Rate utilized the Pearson correlation coefficient as a measure of correlation strength. The analysis revealed a significant positive correlation between scintigraphy and eGFR measurements derived from creatinine levels in children diagnosed with congenital anomalies of the kidney and urinary tract affecting both sides, as illustrated in Figure 2. The Pearson correlation coefficient for this subgroup was 0.71, with a *p*-value of 0.005, indicating a strong and statistically significant correlation. Additionally, for the entire cohort under study, a robust correlation was also observed, with a Pearson correlation coefficient of 0.66 and a *p*-value of less than 0.001, as depicted in Figure 3. These findings underscore the consistency and reliability of renal scintigraphy as a diagnostic tool in evaluating renal function through eGFR measurements across different patient subgroups.

### 3.6. Correlation Between Renal Scintigraphy and Ultrasound Features

**Figure 4:**
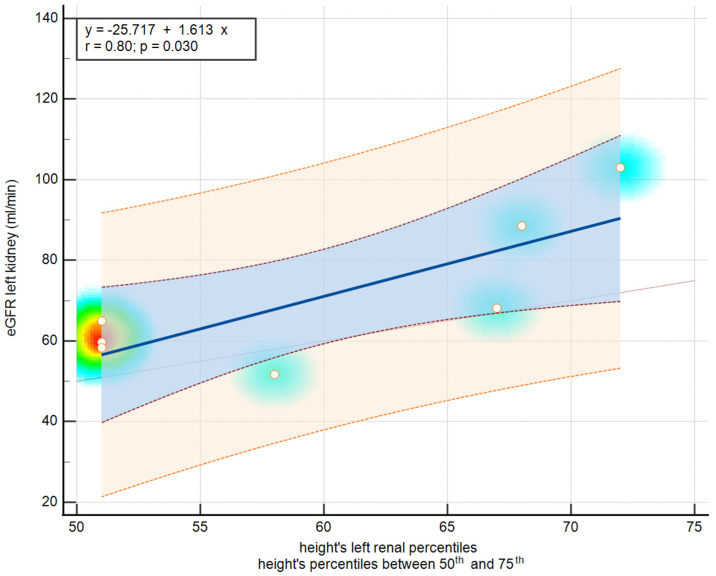
Scatter diagram of correlation left renal scintigraphy and height’s left renal percentiles for height’s percentiles between 50th and 75th—a strong positive linear correlation.

Table 6 and Figure 4 present an analysis of the correlation between renal scintigraphy and various ultrasound features based on body surface area and height percentiles. Among the significant findings, the correlation between the BSA and right renal z-scores for individuals within the 25th–50th percentiles shows a notably negative Pearson correlation (r = −0.64) with a marginally significant *p*-value (0.08). Additionally, a strong negative correlation is observed for the BSA of left renal percentiles within the 25th–50th percentiles, with a Pearson correlation of −0.95, although the *p*-value of 0.20 suggests this might not be statistically significant. On the other hand, the 50th–75th percentiles for left renal percentiles show a substantial positive correlation (r = 0.80) with a statistically significant *p*-value of 0.02. These results indicate variable associations between renal measurements by scintigraphy and ultrasound based on different percentile ranges for BSA and height, warranting further investigation into their clinical implications.

## 4. Discussion

Among the children with CAKUT analyzed in this study, 57.44% were boys, consistent with broader studies that often report a higher dominance of kidney and urinary tract anomalies in males. Similarly, the representation of individuals from rural regions (55.31%) underscores the influence of environmental context, although this specific finding does not directly correlate with CAKUT prevalence. Nevertheless, rural areas may possess certain risk factors (such as exposure to chemicals, infections, or restricted healthcare access) that could potentially contribute to the incidence of CAKUT [26,27]. The median age at diagnosis (24 months) emphasizes the importance of early identification, which can occur either during the antenatal period or at birth. Guidelines recommend performing a renal ultrasound check-up for CAKUT at the first episode of urinary tract infection and newer, but less applicable, recommendations suggest performing an abdominal ultrasound as part of neonatal screening [28].

Scintigraphy examinations at a median age of 112 months provide insights into kidney function. Anthropometric parameters, such as weight and height, aid in comprehending physical growth [29]. Collaborative research across different populations will continue to refine our knowledge and guide effective strategies for managing CAKUT [30,31].

Within the context of CAKUT types, our study reveals a diverse spectrum of anomalies. Renal pelvis and ureter malformations were even more prevalent, accounting for 72.34% of the sample. These anomalies can significantly impact urine flow and kidney function [31,32]. Additionally, vesicoureteral reflux (VUR), present in 27.65% of patients, contributes to progressive nephron injury [33]. Collaborative efforts among pediatricians, obstetricians, and perinatologists are essential for guiding patient care from birth to adulthood. Understanding the evolutionary context and susceptibility of nephrons to hypoxic and oxidative injury will shape future advances in CAKUT management [2,33].

In the pediatric population, defining CKD is challenging due to age-related modifications in GFR during early years of life [34]. In this study, we evaluated the chronic kidney disease staging among children. The findings, as presented in Table 3, reveal the distribution across different CKD stages based on glomerular filtration rate and albuminuria. Notably, all patients fell into the A1 category. While CKD G1 (mild kidney damage with normal or increased GFR) was predominant (80.85%), other stages were also observed, including CKD G2 (8.51%), CKD G3b (4.25%), CKD G4 (4.25%), and CKD G5 (2.12%). These findings emphasize the importance of early detection and monitoring to prevent progression to more severe stages of CKD [34,35]. Underdiagnosis in children may lead to treatment delays and suboptimal clinical management [2]. Furthermore, recent advances in massive-parallel sequencing technology suggest that CKD is more frequent in children and adolescents than previously reported [34].

The analysis critically highlighted that the bedside Schwartz equation significantly underestimated GFR in pediatric CAKUT patients by a mean of −15.5 mL/min/1.73 m^2^, yet it maintained a strong positive correlation with iohexol clearance (r = 0.86). This correlation suggested that the equation could be predictably adjusted for clinical use. In contrast, renal scintigraphy closely mirrored iohexol clearance, with a minimal mean difference of +3.4 mL/min/1.73 m^2^ and a very strong correlation (r = 0.91), indicating its superior reliability and accuracy for estimating GFR in this patient group. These findings indicated that renal scintigraphy should be favored over the Schwartz equation for precise kidney function assessment in pediatric patients with CAKUT, prompting a reassessment of clinical practices to integrate more accurate GFR measurement methods.

Similar studies have also highlighted discrepancies in GFR estimation between scintigraphy and serum creatinine-based methods [7,10,11,36]. These differences can be attributed to various factors such as the accuracy of serum creatinine in reflecting GFR, differences in measurement techniques, and individual patient characteristics [7]. It is important to consider the method of examining the child through scintigraphy. This investigation involves radiation and requires significant co-operation from the child. The child must remain calm while standing during the examination, unlike ultrasound which allows for more movement. It is worth noting that renal scintigraphy provides the sole method for calculating the eGFR for each kidney. Upon summing the two values, the calculated eGFR appears to be greater than the one estimated by creatinine. The negative slope coefficient observed in our study aligns with previous research, indicating the need for caution when comparing GFR values obtained through different estimation methods. These discrepancies underscore the complexity of GFR estimation and the necessity of utilizing multiple approaches to ensure accurate and reliable clinical assessments [7,11,37].

The results indicate a moderate-to-strong correlation between renal scintigraphy and eGFR creatinine in children with CAKUT. The correlation is stronger in children with CAKUT in both sides (r = 0.71, *p* = 0.005), compared to all children included, where the correlation was fairly strong (r = 0.66, *p* < 0.001). These findings are consistent with other studies that have also found a significant correlation between these two measures, reinforcing the utility of renal scintigraphy as a non-invasive method for assessing renal function in pediatric patients [24,38]. However, the strength of the correlation can vary depending on the patient population and the specific methodologies used. In summary, both analyses found that scintigraphy and eGFR were positively correlated, with stronger relationships in certain patient populations. However, the Bland–Altman plot in this study indicated a significant difference between the two methods, suggesting that scintigraphy consistently showed higher values compared to eGFR creatinine. Further research is needed to understand the reasons behind these discrepancies and to determine the most accurate method for estimating GFR in pediatric patients [7,23,24,38,39].

Efficient surveillance of development and prompt interventions are essential to avert and manage growth deficiency in children with CKD [2]. Children may have a significant decrease in height throughout percentiles, particularly during periods of rapid development rates [14,40]. Since height is also utilized in calculating GFR by the bedside Schwartz formula, we sought to see whether it could also be beneficial to assess if adapting it to height or BSA renal percentiles correlates with renal functions [23]. The regression analysis and Pearson correlation coefficient showed a moderate correlation (r= −0.64) between right renal scintigraphy and BSA’s right renal percentiles for BSA between the 25th and 50th percentiles > 75th (r= 0.52). The patients in the BSA’s percentile group between the 25th and 50th for the right kidney were the majority of the double collecting system and, because of the negative correlation, this additional parameter must be taken into consideration when establishing the treatment plan in order to preserve as much as possible of the renal function.

The study also emphasizes compensatory renal hypertrophy, which is a response to renal hypoplasia or agenesis. A moderate association was observed between the scintigraphy findings and the right kidney in the percentile group above the 75th percentile. The patients with renal agenesis or single functioning kidney benefit from early diagnosis by ultrasonography during fetal life, because they can be referred to a pediatric nephrologist or urologist to set up a treatment protocol [16]. Thus, after assessing renal function with DTPA scintigraphy, it is important to also screen the patient for hyperfiltration which leads to renal function decline. It is recommended alongside blood pressure monitoring to check for albuminuria [41].

A good correlation between left renal scintigraphy and height’s left renal percentiles for heights between the 50th and 75th percentiles was obtained, but when we performed the correlation between renal scintigraphy and renal percentiles using BSA, the correlation was weak. This highlights the fact that the nutritional status of the patient must be taken into consideration, and renal percentiles reported to the BSA are likely to be more accurate in this type of patient. This is the element of novelty introduced by this study.

For the BSA percentiles between the 25th and 50th, the eGFR scintigraphy of the right kidney was significantly higher than that of the left kidney (*p* = 0.01), while for the entire lot, the eGFR of the right kidney was significantly higher than that of the left kidney (*p* = 0.02). Another study investigated the accuracy of different equations in evaluating eGFR in a Chinese population with different BMI levels. It found that the accuracy of eGFR estimates varied with BMI, which might be an interesting aspect to consider in relation to our study’s findings [42].

Currently, there has been no research conducted on the link between renal scintigraphy and ultrasound features based on BSA or height’s percentiles. However, the results indicate a potential relationship between these two methods, as evidenced by the observed modifications in the renal parenchyma. While the specific correlation coefficients may vary depending on the patient population and the specific ultrasound features studied, the overall trend indicates that these two methods are related, and further research is needed to determine the clinical implications of these correlations.

### 4.1. Limitations of the Study

Even though this study provided insightful information, there are a few important caveats to note. First of all, the retrospective observational design restricts the capacity to prove causation and raises the possibility of selection bias by default. Moreover, the limited sample size and variability could limit the applicability of the results to larger pediatric CAKUT populations. Furthermore, another limitation of our study is the absence of a control group. This was due to the nature of scintigraphy, an irradiating examination, which is only performed per protocol in selected patients. This protocol is followed when the examination can provide the most relevant information regarding kidney function or confirm a diagnosis. Additionally, the decision not to include cystatin C in the measurement of the estimated glomerular filtration rate in our study was primarily due to the scope and resource allocation of the research. Future studies that address these limitations may yield a more thorough knowledge of the assessment and treatment of pediatric kidney health.

### 4.2. Future Directions

Firstly, carrying out more extensive prospective studies including a variety of patient populations may aid in validating and extending the correlations between various techniques used to evaluate kidney function in children with CAKUT. Investigating the effects of other clinical variables on renal function estimation, such as genetic predispositions, medication use, and dietary determinants, may improve the precision and dependability of juvenile kidney health evaluations. New biomarkers are investigated to be used in association with serum creatinine to assess the kidney function in correlations with renal ultrasound. Collaborative multicenter studies have the potential to enhance data exchange and assist the establishment of standardized methods for the evaluation of kidney health in the context of different types of CAKUT, thereby fostering uniformity and comparability among diverse healthcare environments. Finally, longitudinal follow-up studies that monitor the course of kidney disease and its clinical consequences over time may offer insightful information about the long-term effects of different evaluation techniques and help develop tailored therapy plans for children with CAKUT.

## 5. Conclusions

The study revealed discrepancies between eGFR values obtained through the bedside Schwartz equation, renal scintigraphy, and iohexol clearance, highlighting the complexity of GFR estimation in pediatric patients with CAKUT. By comparing these methods, we found that, while the Schwartz equation significantly underestimated GFR, renal scintigraphy provided closer estimates to iohexol clearance, reinforcing its accuracy and reliability. We started from possible connections between renal scintigraphy and ultrasound characteristics, based on the alterations in renal parenchyma in different CAKUT and patients’ characteristics. The study presented novel insights into the evaluation of CAKUT by uncovering correlations between renal scintigraphy parameters and anthropometric measures, particularly BSA percentiles, indicating the importance of considering additional parameters like the presence of double collecting systems in treatment planning to preserve renal function effectively. Furthermore, the study highlights the significance of compensatory renal hypertrophy, especially in patients with BSA percentiles above the 75th, emphasizing the need for early diagnosis and specialized referral for optimal management protocols.

## Figures and Tables

**Table 1:** Baseline characteristics of the population under study.

Characteristic	Sample Size (*n* = 94)
Male gender (%)	54; (57.44%)
Place of origin from rural areas (%)	52; (55.31%)
Age at the moment of scintigraphy examination (months)	112; (61, 147)
Weight at the moment of examination (kg)	28; (19, 49.25)
Weight percentiles	58; (25, 79.50)
Height at the moment of examination (cm)	136; (110.87, 150)
Height percentiles	65; (30.25, 92.25)
Body surface area (BSA) (m^2^)	1.02; (0.77, 1.43)

For a parametric distribution, the results are presented as mean ± standard deviation, whereas for a non-parametric distribution, the results are presented as median and interquartile range (IQR).

**Table 2:** CAKUT types and other associated pathologies.

Anomaly or Pathology (CAKUT)	Sample Size (*n* = 94)
**Kidney anomalies**	
Renal hypoplasia	12 (12.77%)
Solitary kidney	4 (4.26%)
Polycystic kidney disease	6 (6.38%)
Renal dysplasia	6 (6.38%)
‘Horseshoe’ kidneys	2 (2.12%)
Duplex Kidney (Duplicated collecting system)	16 (17.02%)
**Collecting system anomalies**	
Ureteropelvic junction stenosis	32 (34.04%)
**Bladder anomalies**	
Vesicoureteral reflux *	26 (27.65%)
**Urethral anomalies**	
Posterior urethral valve	8 (8.51%)

* Includes patients with primary and secondary vesicoureteral reflux; the percentages expressed are from the patients with vesicoureteral reflux.

**Table 3:** Chronic kidney disease staging according to GFR and albuminuria.

Stages	Sample Size (*n* = 94)
CKD G1	76 (80.85%)
CKD G2	8 (8.51%)
CKD G3a	0
CKD G3b	4 (4.25%)
CKD G4	4 (4.25%)
CKD G5	2 (2.12%)
CKD A1	94 (100%)

**Table 4:** Comparison of GFR measurement methods.

GFR Measurement Method	Mean GFR (mL/min/1.73 m^2^)	Standard Deviation (SD)	Mean Difference vs. Iohexol (mL/min/1.73 m^2^)	*p*-Value for Difference	Correlation with Iohexol GFR (r)	*p*-Value for Correlation
Iohexol Plasma Clearance	129.7	53.8	N/A	N/A	N/A	N/A
eGFR from Schwartz Equation	114.2	42.3	−15.5	0.023	0.86	<0.001
GFR from Renal Scintigraphy	133.1	56.5	3.40	0.57	0.91	<0.001

**Table 5:** Independent sample t-tests between eGFR scintigraphy in each kidney.

BSA’s Percentiles	Right Kidney Scintigraphy	Left Kidney Scintigraphy	*p* Value
<25th	57.45 ± 37.75	43.90 ± 40.93	0.24
25th–50th	102.02 ± 41.52	35.60 ± 26.05	0.01
50th–75th	79.18 ± 31.14	74.50 ± 19.02	0.71
>75th	81.53 ± 40.58	65.89 ± 33.66	0.23
Entire lot	76.03 ± 40.91	57.46 ± 35.91	0.02

**Table 6:** Correlation between renal scintigraphy and ultrasound features by BSA and height.

Category	Feature	Pearson Correlation (r)	*p* Value
**Right Renal Z Score (BSA)**	<25th Percentile	0.33	0.16
	25th–50th Percentiles	−0.64	0.08
	50th–75th Percentiles	−0.40	0.28
	>75th Percentile	0.52	0.08
**Right Renal Z Score (Height)**	<25th Percentile	0.15	0.54
	25th–50th Percentiles	−0.10	0.84
	50th–75th Percentiles	0.41	0.30
	>75th Percentile	0.16	0.54
**Left Renal Z Score (BSA)**	<25th Percentile	0.33	0.19
	25th–50th Percentiles	−0.95	0.20
	50th–75th Percentiles	0.37	0.35
	>75th Percentile	−0.34	0.15
**Left Renal Z Score (Height)**	<25th Percentile	0.42	0.09
	25th–50th Percentiles	0.18	0.81
	50th–75th Percentiles	0.80	0.02
	>75th Percentile	−0.29	0.20

## Data Availability

The information is contained within this article in its entirety. For additional information, please feel free to inquire with either the original author or the corresponding author. The public’s access to the data is restricted as a result of the patient privacy standards that regulate the handling of clinical data.
